# Modifying Beauty Is Not Priceless: A Rare Case of Silicone-Induced Hypercalcemia With Renal Failure

**DOI:** 10.7759/cureus.25890

**Published:** 2022-06-13

**Authors:** Nusha Fareen, Mohammad Umair Zafar, Zaka Ahmed, Mohammad A Hossain, Sushmitha P Diraviam, Sobaan Taj, Avais Masud

**Affiliations:** 1 Internal Medicine, Jersey Shore University Medical Center, Neptune City, USA; 2 Nephrology, Jersey Shore University Medical Center, Neptune City, USA; 3 Medicine, Hackensack Meridian School of Medicine, Nutley, USA; 4 Internal Medicine, St. George’s University, West Indies, GRD

**Keywords:** vitamin d, renal failure, granulomatous inflammation, silicone, hypercalcemia

## Abstract

Silicone implant-induced hypercalcemia is a rather rare pathological entity. There are only a few published reports on the topic. Here, we have reported a case of acute kidney injury in the background of hypercalcemia and elevated vitamin D level in a transgender patient with a history of silicone injections in the breast and buttocks for cosmetic purposes.

## Introduction

Silicone and polymethylmethacrylate injections for breast, buttock, and leg augmentation have been regularly utilized for decades [1,2]. Food and Drug Administration (FDA), USA, although approves the use of silicone implants for cosmetic purposes or for reconstructive purposes following breast surgery following cancer, trauma, or other congenital conditions, the approval comes with a caution related to silent rupture [3]. Granulomatous hypercalcemia following silicone implant or injection is a rather rare entity [4-6]. Hypercalcemia is the most common presentation of an underlying condition. Literature search reveals only a few such reported cases in female, transgender, and male patients following receiving silicone implants or injections or even topical preparations for cosmetic purposes [4-14].

Here, we have reported a case of acute kidney injury in the background of hypercalcemia and elevated vitamin D levels in transgender women with a past history of silicone implant, with histopathological findings of acute and chronic tubulointerstitial nephropathy with abundant calcium and phosphate deposits (nephrocalcinosis). To the best of our knowledge, ours is the first case report with documented evidence of nephrocalcinosis in the background of hypercalcemia in a transgender patient with past history of silicone injections for cosmetic purposes. The patient was managed successfully with steroids, bisphosphonates, hydration, and surgical removal of silicone implants of breast.

## Case presentation

A 31-year-old transgender patient who currently identifies as a female with a past medical history significant for hormone replacement treatment with estradiol and silicone injections breast and buttocks augmentation presents to the emergency department for abnormal reports from a routine outpatient workup. The patient, at the time of presentation, complained of severe frontal headache, nausea, non-bilious and non-bloody vomiting, and intermittent blurred vision. The patient’s vitals were as follows: body temperature was 98.5°F, blood pressure was 145/90 mm Hg, and pulse was 71 beats per minute (bpm). Physical examination was unremarkable. Laboratory investigations were significant for creatinine level of 2.66 mg/dL (reference range: 0.8-1.2 mg/dL) and elevated calcium level of 13.6 mg/dL (reference range: 8.4-10.2 mg/dL) (Table 1). Further evaluation showed high angiotensin-converting enzyme (ACE) level of 171 U/L (reference range: 9-67 U/L), 1.25 dihydroxy vitamin D level of 173 pg/mL (reference range: 19.9-79.3 pg/mL), low parathormone (PTH) level of 7.6 pg/mL (reference range: 12-88 pg/mL) and high parathormone-related protein (PTHrp) at 5.6 pmol/L (reference range: 0.0-2.3 pmol/L). Electrocardiogram (ECG) showed sinus rhythm with first-degree atrioventricular (AV) block. The computed tomography (CT) chest demonstrated increased soft tissue density and innumerable nodular lesions within bilateral breast tissue and subcutaneous fatty tissue of the anterior chest wall. The right kidney is malrotated and not placed in the right iliac fossa. A kidney ultrasound confirmed the absence of hydronephrosis. Serum protein electrophoresis was negative for paraproteinemia The patient was treated with Intravenous (IV) fluid, bisphosphonates, and calcitonin. Once calcium and renal function tests (including creatinine level of 1.7 mg/dL) improved, she was discharged on daily oral prednisone, 5 mg.

**Table 1:** Laboratory parameters at the time of first admission

Test	Result	Unit	Normal range
Parathyroid hormone	7.6	pg/mL	12-88
Parathyroid hormone-related protein	5.6	pmol/L	0.0-2.3
Thyroid-stimulating hormone	0.606	uIU/mL	0.300-4.500
1, 25 dihydroxy vitamin D	173	pg/mL	19.9-79.3
Calcium	13.6	mg/dL	8.5-10.5
Angiotensin-converting enzyme (ACE)	172	U/L	9-67
Erythrocyte sedimentation rate	16	mm/h	0-15
Creatinine	2.66	mg/dL	0.61-1.24
Blood urea nitrogen	25	mg/dL	5-25

**Figure 1:**
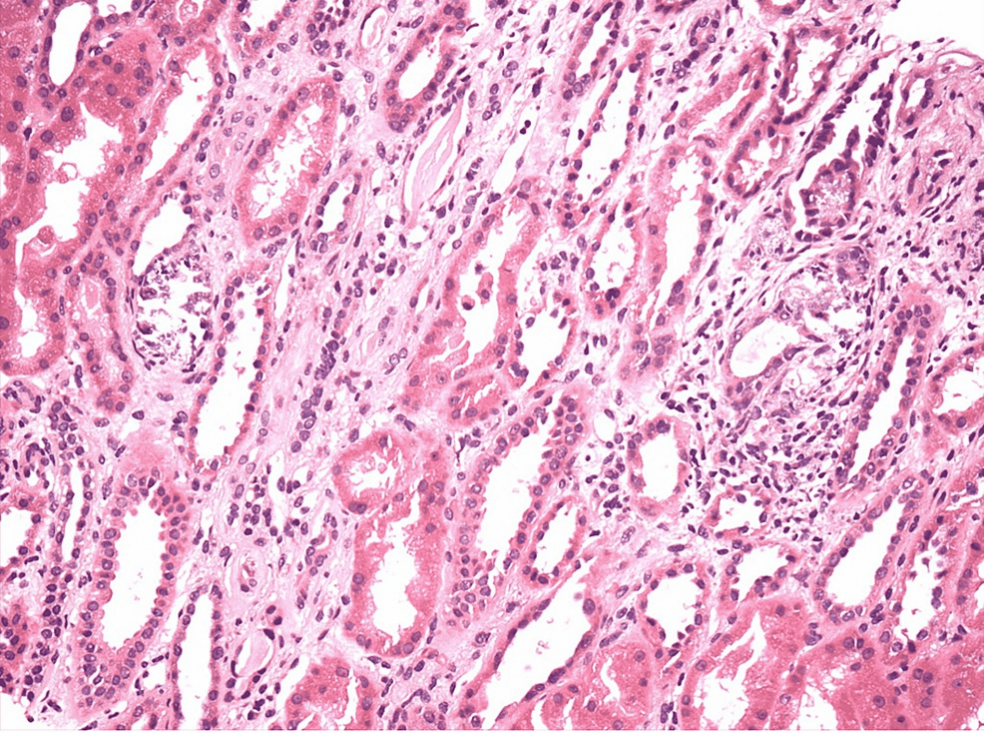
Interstitial inflammation composed mainly of lymphocytes and vessels exhibit mild arteriosclerosis.

**Figure 2:**
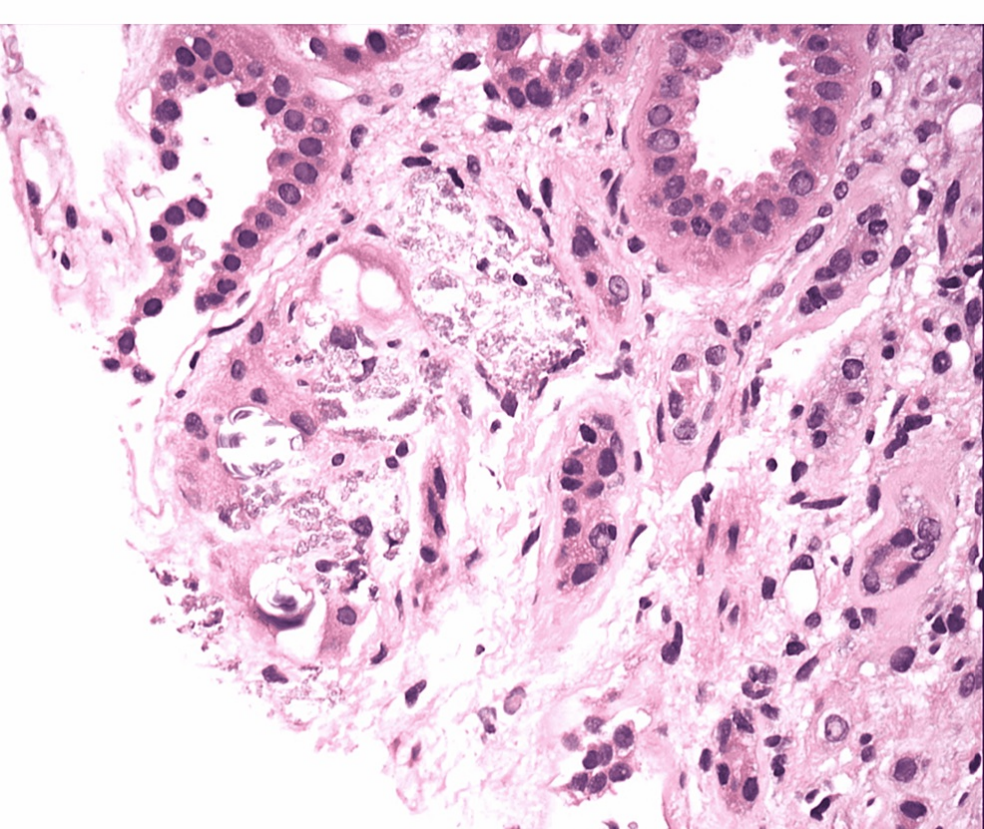
Tubular and focal interstitial calcification, consistent with calcium phosphate.

The patient was readmitted two weeks later with continued abnormal blood work in the outpatient setting. Upon admission, calcium level was elevated at 14 mg/dL and creatinine level was elevated at 1.88 mg/dL. The patient at this time had complaints of mild confusion, polyuria, and lower abdominal cramping and no history of headaches, blurry vision, nausea, vomiting, constipation, or fatigue. On inquiry, she complained to have bowel movements with streaks of blood the previous day. The patient’s vital signs and physical examination were unremarkable. Urine analysis revealed hematuria and EKG revealed low voltage QRS compared to the previous EKG. Hypercalcemia was managed conservatively with IV fluids and zoledronic acid. Renal biopsy was performed during this admission to rule out underlying renal pathology for hypercalcemia. Histopathological examination revealed acute & chronic tubulointerstitial nephropathy with abundant tubular calcium phosphate deposits consistent with nephrocalcinosis (Figures 1, 2).

Bilateral diagnostic mammogram revealed extremely dense breasts with extensive silicone injections throughout both breasts extending into the axilla bilaterally. Breast MRI was not recommended due to probable silicone granulomas throughout the breasts. Subsequently, bilateral silicone breast implants were removed; however, the patient continues to have hip implants. The patient continues to get estradiol injections and is being monitored on an outpatient basis with 20 mg of prednisone and ketoconazole. Her calcium levels have stabilized around 12 mg/dL on follow-up.

## Discussion

Silicone breast implants contain an outer covering called elastomer covering silicon in gel form; elastomer protects the inner silicone gel from contracture and rupture [1,4,6]. Despite the protection offered by breast implants, implant rupture is common, especially following mammography or any other traumatic events. Following rupture of the elastomer and release of silicone gel, the said gel may reach different other distant body parts through the lymphatic system or blood circulation.

The host immune system can trigger foreign body reactions against the ruptured silicone gel, leading to a granulomatous condition affecting different organs. Several studies have reported that the prevalence of sarcoidosis is quite high among women with silicone breast implants compared to women without any such implants [1,12].

Ruptured silicone-induced immunological reactions usually have a latent period of acute inflammatory reaction (which might last for several years) followed by delayed granulomatous reaction. In most of such cases, initial presenting features include hardening of overlying skin and soft tissue along with formation of lumps and nodules which might lead to ulcerations [1,12].

Silicone-induced immunological reactions lead to the formation of siliconomas. The first case of siliconoma was described by Winer et al in the year 1964 [13]. In this case study, the researchers reported three cases of tissue reactions following liquid silicone injections. Literature search reveals that siliconomas leading to hypercalcemia are quite rare; there are few such cases reported so far.

In one of the initially reported cases, Kozeny et al. reported hypercalcemia in a transgender patient of 33 years [4]. The said patient received silicone injections in breast, buttock, and face for cosmetic purposes. The patient was managed with steroids and hydration. However, after stopping steroid therapy calcium level again increased like in our case [4]. In another case, Ogbuagu et al. presented a similar case of hypercalcemia in a 38 years old female patient who received silicone breast implants and silicone injections in buttocks [5]. Interestingly, in a 72-year-old lady, topical silicone with ultrasound therapy for facial wrinkle removal led to hypercalcemia which was successfully managed with steroid therapy [6]. In another similar case report, Agrawal et al. reported hypercalcemia in a transgender patient receiving bilateral silicone breast implants [7]. Several other case reports also documented similar findings in female, transgender, and male patients [8-11]. Some of the patients, not all, responded well with correction of electrolyte imbalance (hydration) and steroids with or without administration of bisphosphonates [6,7,11]. Surgical removal of silicone granuloma was reported in two of the cases [8,9].

Here, we have reported a similar case in a 31-year-old transgender patient with a past history of silicone injections for breast and buttock augmentation. Besides hypercalcemia, she presented with elevated PTHrp, vitamin D3, ACE, creatinine, thyroid-stimulating hormone (TSH), and erythrocyte sedimentation rate (ESR). Elevated creatinine and high normal blood urea nitrogen (BUN) suggested acute kidney injury. The patient was successfully managed with fluid replacement, bisphosphonates, and surgical removal of silicone breast implants. At the time of writing the case report, removal of hip implants was planned. Renal biopsy was done to rule out underlying malignancy and explore the cause of acute kidney injury. HP examination revealed acute and chronic tubulointerstitial nephropathy with abundant deposits of calcium and phosphate most likely related to hypercalcemia in this patient. Among all the reported cases, our case is the first reported case of nephrocalcinosis occurring in the background of hypercalcemia in a transgender patient with silicone breast and hip implants.

The exact underlying mechanism of silicone implants or injections induced hypercalcemia is not yet known. Foreign body granuloma formation following silicone implant rupture or injection might have a possible role in precipitating hypercalcemia. It is already established that granulomatous conditions like sarcoidosis can lead to calcitriol-induced hypercalcemia. In these conditions, macrophages release 1-alpha-hydroxylase which facilitates the conversion of inactive vitamin D to its active form. Calcitriol has an immunomodulatory action resulting in decreased activity of T cells, most likely due to inhibition of IL-2 and γ-interferon [14]. Moreover, in vitro immunohistochemical studies have demonstrated the participation of macrophages in sarcoidosis and foreign body granuloma through elicitation of 1-alpha-hydroxylase [14,15].

Under normal circumstances, renal 1-alpha-hydroxylase is responsible for the production of an active form of vitamin D, calcitriol. High circulating calcitriol elicits negative feedback on 1-alpha-hydroxylase production and thus inhibits further calcitriol production. This regulatory pathway is lacking in macrophages, leading to uncontrolled calcitriol production [13]. Another mechanism of increased calcitriol has been reported to be due to estrogen treatment though the mechanism is unclear [16]. Unregulated and excessive production of calcitriol can lead to increased intestinal absorption of calcium and bone resorption leading to hypercalcemia and subsequent nephrocalcinosis. 

Till now there is no established definitive treatment for silicone implant-induced hypercalcemia. Steroids might have some role in correcting hypercalcemia; however, they are not as effective as in sarcoidosis. Still, in many of the reported cases including ours, steroid therapy along with the removal of silicone implants improved the condition. However, side effects associated with prolonged steroid therapy should be kept in mind. Another possible treatment option is ketoconazole, which is an inhibitor of vitamin D-24 hydroxylase. There have been some reports of using Ketoconazole as a means to reduce serum 1,25-dihydroxy vitamin levels [17,18]. The use of ketoconazole has helped to stabilize this patient’s calcium levels; however, it remains elevated at 12 mg/dL which we hypothesize is likely due to remaining implants in the hip region.

## Conclusions

Physicians should always consider silicone-induced hypercalcemia and nephrocalcinosis in patients with past history of receiving silicone implants, injections, or even topical preparations presenting with a high level of calcium or local changes at the site of silicone injections or implants (like breast tissue, facial tissue, buttocks, etc.). Early diagnosis, steroid therapy, correction of hydration, bisphosphonate therapy along with the removal of silicone implants are crucial steps in the management of this condition.
